# Lipoarabinomannan Antigen Assay (TB-LAM) for Diagnosing Pulmonary Tuberculosis in Children with Severe Acute Malnutrition in Mozambique

**DOI:** 10.1093/tropej/fmaa072

**Published:** 2020-10-10

**Authors:** Dulce-Vasco Osório, Isabelle Munyangaju, Argentina Muhiwa, Edy Nacarapa, Amancio-Vicente Nhangave, Jose-Manuel Ramos

**Affiliations:** 1 Department of General Medicine, Macia Health Center, Gaza, Mozambique 0000; 2 Elizabeth Glaser Pediatric AIDS Foundation, Maputo, Mozambique 0000; 3 Department of Internal Medicine, Carmelo Hospital, Gaza, Mozambique 0000; 4 Provincial Health Directorate, Gaza Provincial Research Nucleus, Gaza, Mozambique 0000; 5 Department of Internal Medicine, University General Hospital of Alicante and University Miguel Hernandez de Elche, Alicante, Spain

**Keywords:** urine lipoarabinomannan antigen, tuberculosis, children, severe acute malnutrition, Mozambique

## Abstract

**Background:**

Tuberculosis (TB) and malnutrition are important causes of morbidity and mortality in children in the developing world.

**Aims:**

To assess the prevalence of pulmonary TB in severely malnourished children and evaluate TB detection using the urine lipoarabinomannan antigen assay (TB-LAM).

**Methods:**

A retrospective analysis was conducted in all pediatric inpatients with severe acute malnutrition at a rural health center in Mozambique, from February to August 2018. All children underwent a physical examination and chest X-ray, and their nasopharyngeal aspirates and stool specimens were studied for mycobacterial culture and subjected to the Xpert MTB/RIF assay. TB-LAM tests were performed on urine.

**Results:**

Of 45 included cases, 17 (37.8%) were clinically diagnosed as pulmonary TB. None of these were detected by the Xpert MTB test; 4 (8.9%) nasopharyngeal aspirates were TB-culture positive. Seventeen patients (37.8%)—all clinically diagnosed with TB—tested positive on the TB-LAM, while 23 (51.1%) were negative. In 5 (11.1%), the urine LAM was not done.

**Conclusion:**

Although our sample size was small, TB was diagnosed and treated in more than a third of included children. The urine TB-LAM test showed a perfect correlation with clinical diagnosis of childhood TB.

**LAY SUMMARY:**

Severe acute malnutrition makes children more vulnerable to tuberculosis (TB) infections, but it is difficult to detect TB in children because they cannot always cough up phlegm, which is used in diagnostic processes. This study aimed to find out how many severely malnourished children had TB in Gaza, Mozambique, and to test the accuracy of a less-used diagnostic test: the lipoarabinomannan assay (TB-LAM). Of the 45 severely malnourished children who were admitted to our hospital, 17 were diagnosed with TB by their doctor. The TB-LAM corroborated the clinical diagnosis in all cases, while the other tests (Xpert MTB/RIF assay) and cultures failed to detect most of them. Overall, more than a third of severely malnourished children had TB, and the TB-LAM test—a simple, point-of-care method—was a highly accurate way to diagnose them. While larger studies are needed to confirm these results, our findings suggest that the TB-LAM could vastly improve TB diagnosis in malnourished children.

## INTRODUCTION

Tuberculosis (TB) and malnutrition are important causes of morbidity and mortality in children in the developing world. In 2018, an estimated 1.1 million children developed TB, and 170 000 died [1]. At the same time, about 50 million children suffer from severe wasting, resulting in nearly 1 million annual deaths, mostly in sub-Saharan Africa and Asia [2].

Severe acute malnutrition (SAM) is associated with serious lower respiratory tract infections such as TB and pneumonia, and its immunosuppressive effect facilitates the rapid progression from TB infection to active disease [3], further complicating this diagnosis in children. In 2013 in Mozambique, about 43.0% of all children aged under 5 years were chronically malnourished [4] and 13.2% of the population had HIV/AIDS [5]—one of the highest prevalence estimates in the world, with serious implications for children.

Diagnosing childhood TB remains challenging because young children are unable to expectorate sputum, and most have paucibacillary TB forms. The gold standard for diagnosis is based on detecting *Mycobacterium* *tuberculosis* (MTB). However, due to the paucibacillary nature of childhood TB, microbiological diagnosis is extremely difficult [6]. Currently, the Xpert MTB/RIF diagnostic test is recommended for this population [7, 8], but the LAM assay is another possibility, detecting the presence of the *M. tuberculosis* cell wall antigen LAM in urine and other body fluids at the point-of-care and providing results within 25 min [9, 10]. While the World Health Organization (WHO) recommends the TB-LAM in patients with HIV infection and a CD4 count of less than 100 cells/ml^3^ [11], there is limited experience in children and less in malnourished children [7].

We aimed to assess the prevalence and characteristics of pulmonary TB in children with SAM and assess the urine LAM antigen test for detecting TB.

## METHODS

### Study design and setting

This retrospective, cross-sectional study collected data from patient histories, plus ward and discharge registers for all children aged 0–59 months admitted to the pediatric inpatient unit of Macia Health Center, in Gaza Province, Mozambique, between 1 February and 31 August 2018. In a recent study performed in Chokwe Carmelo Hospital-Daughters of Charity, Saint Vincent of Paul, Gaza Province, among children with TB less than 15 years of age and adults with TB 15 years of age or more, 62% and 82.8%, respectively, were co-infected with HIV [12, 13].

### Definitions

The National Program for the Control of Tuberculosis of Mozambique defines the criteria for clinical diagnosis of pulmonary TB as (i) signs/symptoms: (a) persistent cough (>2 weeks), unremitting cough; (b) weight loss/failure to thrive; (c) persistent (>1 week), unexplained fever reported by guardian; (d) persistent, unexplained lethargy or reduced playfulness; (e) infants 0–60 days with additional signs and symptoms like neonatal pneumonia, unexplained hepatosplenomegaly or sepsis-like illness; (ii) findings on chest X-ray congruent with pulmonary TB (presence of lymphadenopathy and/or abnormalities consistent with TB as new infiltrates) and read by two blinded operators (clinician and TB expert); (iii) history of exposure to *M. tuberculosis* within the preceding 12 months; or (iv) response to antituberculosis treatment yet no acid-fast bacillus on the sputum smear or a negative Xpert MTB/RIF test. To classify TB, we used Graham, *et al.*’s [14] revised classification of intrathoracic TB case definitions for diagnostic evaluation studies in children.

We included all children with SAM (weight for height SD SCORE less than −3, or the presence of symmetrical pitting edema) according to WHO Child Growth Standards, in the study period.

All children were evaluated by a medical doctor for clinical diagnosis, underwent nasopharyngeal aspiration for GeneXpert and *M. tuberculosis* culture, submitted feces specimens for GeneXpert and urine for TB-LAM testing, and received a chest X-ray. To detect TB-LAM mycobacterial antigens in urine, a lateral flow assay for TB-LAM (Alere Determine TB LAM Ag, Abbott Laboratories, Chicago, IL, USA) was used. The GeneXpert assay used was the Xpert MTB/RIF (version 4.3; Cepheid, Sunnyvale, CA, USA).

The attending physicians making the clinical-radiological diagnosis were blinded to the result of the TB-LAM, while the laboratory technicians were blinded to the clinical diagnosis.

### Data analysis

Clinical and microbiological variables were compared according to clinical TB diagnosis, using the Fisher’s exact test (categorical variables) or Student’s *t*-test (continuous variables). *p*-Values of less than 0.05 were considered significant.

### Ethical considerations

The Mozambican National Bioethics Committee for Health approved the study protocol (IRB00002657).

## RESULTS

Forty-five children with SAM were admitted to the ward; 17 (37.8%) had symmetrical edema; 20 (44.4%) had both symmetrical edema and weight for height SD SCORE ˂−3; and 8 (17.8%) weight for height SD SCORE ˂−3. Seventeen (37.8%) of these children were clinically diagnosed with TB. Table 1 shows their demographic and clinical characteristics according to TB status. Fever, weight loss and failure to thrive were statistically associated with clinical diagnosis of pulmonary TB, as were weight for height SD score ˂−3 and symmetrical edema (combination), and weight for height SD score ˂−3 (isolated). Mean height and mid-upper arm circumference were significantly lower in children with pulmonary TB. Seventeen (37.8%) chest X-rays were consistent with TB, of which 16 (94.7%) cases had been clinically diagnosed (*p* < 0.001).

**Table 1:** Demographic and clinical characteristics of children with severe acute malnutrition, according to clinical diagnosis of TB

Variables	Total (*N* = 45)	TB (*N* = 17)	No TB (*N* = 28)	*p*-Value
Demographic variables				
Age (months), mean (SD)	17 (5.0)	16.1 (5.2)	17.9 (4.8)	0.23
Girls, *n* (%)	21 (46.7)	8 (47.1)	13 (46.4)	0.96
Weight (kg), mean (SD)	7.5 (1.6)	7.0 (1.7)	7.8 (1.5)	0.99
Length (cm), mean (SD)	73.1 (5.6)	71.2 (3.7)	74.4 (4.6)	**0.05**
MUAC (cm), mean (SD)	11.6 (2.1)	10.6 (2.4)	12.3 (1.6)	**0.01**
Weight for height SD score, *n* (%)				0.15
<−3	17 (37.8)	8 (47.1)	9 (32.1)	
−3 to <−1	12 (26.7)	6 (35.3)	6 (21.4)	
≥−1	16 (35.6)	3 (17.6)	13 (46.6)	
Weight for height SD score ˂−3 isolated, and with symmetrical edema malnutrition, *n* (%)	28 (62.2)	14 (82.4)	14 (50.0)	**0.03**
HIV status, positive, *n* (%)	10 (22.0)	6 (35.0)	4 (14.0)	0.14
Clinical variables, *n* (%)				
Cough	27 (60.0)	12 (71.0)	15 (54.0)	0.35
Fever	21 (47.0)	12 (71.0)	9 (32.0)	**0.02**
Loss of weight	28 (62.0)	14 (82.0)	14 (50.0)	**0.05**
Reduced playfulness	27 (60.0)	14 (82.0)	13 (46.0)	**0.02**
Lethargy	43 (96.0)	17 (100.0)	26 (93.0)	0.99
Edema	36 (80.0)	13 (77.0)	23 (82.0)	0.17
Diarrhea	9 (20.0)	6 (21.4)	3 (17.6)	0.99
Other diagnostic procedures, *n* (%)				
Chest X-ray consistent with TB	17 (37.8)	16 (94.1)	1 (5.9)^a^	**<0.001**

MUAC, mid-upper arm circumference; SD, standard deviation; TB, tuberculosis.

a The one child with chest X-ray compatible with TB and with severe acute malnutrition was decided not to have TB as a diagnosis of bronchopneumonia was made, which improved after a 7-day course of antibiotic treatment as per the national pediatric TB diagnostic algorithm.

The 45 nasopharyngeal aspirates and 45 stool samples processed (one of each specimen per child) with the Xpert MTB/RIF assay were negative. Four of 45 (8.9%) of the nasopharyngeal aspirates (one per child) cultured positive for *M. tuberculosis*; this represents 23.5% of those with clinical signs and symptoms consistent with TB vs. 0/28 of those without a clinical diagnosis of TB (*p* < 0.001). Of the 45 cases, 17 (37.8%) tested positive on the TB-LAM (all of the clinically diagnosed cases), 23 (51.1%) tested negative; while in 5 (11.1%) the urine LAM was not done.

## DISCUSSION

The prevalence of pulmonary TB among children with SAM in our study was 37.5%—higher than the range observed elsewhere, from 1.6% in malnourished children in Zambia to 22.8% in southern Ethiopia [15–17]. One of the reasons for this difference could be the high TB prevalence in Mozambique, one of the countries with the highest TB burdens in the world [1]. We also found a high prevalence of HIV in these malnourished children. Taken together, the high prevalence of TB countrywide and the high prevalence of HIV in our sample could possibly explain the high rate of TB we found in the children in our study.

Other relevant results include the perfect correlation between positive TB-LAM tests and clinical diagnosis of TB. Indeed, the TB-LAM assay performed significantly better than the Xpert MTB/RIF assay. These results corroborate previous studies suggesting that the TB-LAM by Alere Determine TB LAM Ag, as in our study, is more useful than the Xpert MTB/RIF in children, especially in the presence of malnutrition or HIV [7, 8]. This study shows much higher sensitivity than any of the previous studies [7, 8], probably due to the characteristics of this study population (all with SAM); the other studies were performed in children in whom TB was suspected but not specifically in malnourished children. However, other studies have reported contrasting results, determining that urine TB-LAM testing was not apt for intensive case finding in low-income countries [6, 18]. Further research is needed to clarify the utility of urine TB-LAM testing in these populations.

The study has several limitations: first is its small sample size, which reduces its generalizability. Second, this was a retrospective study so we cannot rule out some misclassification or information bias. Third, the TB-LAM was done in just 89.9% of the cases, not in all of them. Finally, there was no control group, for example, children admitted with symptoms of TB but without SAM who were evaluated in the same way.

Small sample size notwithstanding, over a third of the children with SAM were diagnosed and treated for TB. The urine TB-LAM test was positive in all clinically diagnosed cases, thus improving the potential for disease detection. Additional research on the TB-LAM test is needed in both malnourished and non-malnourished children.

In conclusion, considering the low cost and rapid, point-of-care design of the TB-LAM test, this assay is a potential game changer for diagnosing TB in HIV-infected and malnourished children.
